# Effects of dietary supplements on cognitive outcomes and physiological biomarkers in mild cognitive impairment: a systematic review and network meta-analysis

**DOI:** 10.3389/fnut.2026.1775177

**Published:** 2026-04-29

**Authors:** Xiaolong Wang, Shan Jiang, Tianle Zou, Shuhui Shang, Enming Zhang, Lan Rong, Qiong Fang

**Affiliations:** 1 Department of Geriatrics, Ruijin Hospital, Shanghai Jiao Tong University School of Medicine, Shanghai, China; 2 School of Nursing, Shanghai Jiao Tong University, Shanghai, China

**Keywords:** cognitive function, dietary supplements, elderly, mild cognitive impairment, network meta-analysis, systematic review

## Abstract

**Background:**

Mild cognitive impairment (MCI) represents a transitional stage between normal aging and dementia, affecting a significant proportion of the elderly population. Non-pharmacological nutritional supplements, such as vitamins, omega-3 fatty acids, probiotics, and herbal extracts, have been proposed as potential interventions to mitigate cognitive decline and improve physiological biomarkers. However, evidence on their efficacy remains inconsistent.

**Objective:**

This systematic review and network meta-analysis (NMA) aimed to evaluate the effectiveness of various non-pharmacological nutritional supplements on cognitive function and key physiological indicators (e.g., BDNF, Aβ42, Aβ40) in elderly individuals with MCI.

**Methods:**

This systematic review followed PRISMA-NMA guideline and was registered in PROSPERO (CRD420251079079). We searched PubMed, Embase, Cochrane Library, Web of Science, and CNKI databases from inception to June 2025 for randomized controlled trials (RCTs) comparing dietary supplements to placebo or no intervention. Data extraction included cognitive scores (e.g., MMSE, MoCA, FSIQ) and physiological markers. Risk of bias was assessed using the Cochrane Risk of Bias Tool. Pairwise meta-analyses and NMA were conducted using random-effects models, with standardized mean differences (SMD) for continuous outcomes. Heterogeneity was assessed via *I*^2^ statistics, and sensitivity analyses were performed to test robustness.

**Results:**

Thirteen trials involving 2,451 participants were included. PUFA supplements showed the greatest cognitive benefit (*SMD* 0.91; *95% CI* 0.21–1.61) and ranked first according to SUCRA values. Supplementation significantly reduced Aβ42 levels, while effects on BDNF and Aβ40 were non-significant. Heterogeneity was substantial (*I*^2^ = 96%), and sensitivity analyses demonstrated attenuated effect sizes after removing studies at high risk of bias.

**Conclusion:**

Dietary supplements may offer potential cognitive benefits in MCI, but evidence is limited by study quality and heterogeneity. High-quality RCTs are needed to confirm these findings.

**Systematic review registration:**

Identifier CRD420251079079.

## 1 Introduction

Mild cognitive impairment (MCI) is a clinical syndrome characterized by cognitive decline that exceeds age- and education-related expectations but does not yet significantly compromise independence in daily functioning (1). MCI is regarded as an intermediate stage between normal aging and dementia, with amnestic MCI frequently progressing to Alzheimer’s disease (AD), whereas non-amnestic MCI may evolve into vascular dementia, frontotemporal dementia, or other subtypes (2). Epidemiological studies indicate that the prevalence of MCI among individuals aged ≥65 years ranges from 15 to 20% and increases steadily with age (3), with an annual conversion rate to dementia of approximately 10–15% (4). According to United Nations projections (5), the global population aged ≥65 years is expected to reach 2.47 billion by 2,100, representing more than one-quarter of the world’s population. With the accelerating pace of population aging, MCI and its progression have emerged as increasingly critical public health challenges.

Current pharmacological treatments for MCI, such as cholinesterase inhibitors and memantine, have shown limited effectiveness in delaying disease progression and are frequently associated with adverse effects, which contribute to poor patient adherence (6). These limitations have shifted clinical and research interest toward non-pharmacological interventions, particularly dietary supplements. Such interventions are highly accessible, cost-effective, and generally well tolerated, and include vitamins (e.g., B-vitamins, vitamin D) (7), omega-3 polyunsaturated fatty acids (PUFAs) (8), probiotics, and herbal extracts (9). Mechanistically, these supplements may exert neuroprotective effects through several pathways: B-vitamins regulate homocysteine metabolism and reduce its neurotoxicity (10); *ω*-3 PUFAs exhibit anti-inflammatory properties, enhance synaptic plasticity, and increase brain-derived neurotrophic factor (BDNF) levels (8); and probiotics modulate gut microbiota composition, reduce systemic inflammation, and influence gut–brain axis signaling (11).

During the onset and progression of MCI, several physiological biomarkers, including reduced BDNF levels (reflecting impaired neurogenesis) (12), elevated amyloid-β peptides (Aβ42 and Aβ40), and abnormal oxidative stress markers (13), serve as objective indicators for evaluating intervention efficacy. Preclinical studies and observational evidence suggest that dietary supplements may beneficially modulate these biomarkers. For example, *ω*-3 supplementation has been associated with increased BDNF expression and reduced Aβ deposition in animal models (14).

Despite these mechanistic insights, clinical evidence remains inconsistent. Multiple systematic reviews and meta-analyses have reported conflicting findings: whereas some studies indicate beneficial effects of *ω*-3 PUFAs and B-vitamins on cognitive outcomes, several high-quality randomized controlled trials (RCTs) have failed to demonstrate significant benefits (14). Moreover, existing reviews seldom provide systematic comparisons across supplement categories and rarely employ network meta-analysis (NMA) to rank the relative effectiveness of different interventions (15).

Therefore, this study conducted a systematic review and NMA to synthesize RCT evidence regarding the effects of various non-pharmacological dietary supplements on cognitive function and physiological biomarkers in older adults with MCI. By enabling indirect comparisons and generating treatment rankings, the NMA aims to inform clinical decision-making and guide future research priorities.

## 2 Methods

This systematic review and network meta-analysis was conducted in accordance with the PRISMA extension statement for network meta-analyses (PRISMA-NMA) (16). The reporting checklist is provided in Appendix 1. The protocol was registered in PROSPERO (CRD420251079079).

### 2.1 Search strategy

A comprehensive search was conducted in the following English and Chinese electronic databases from inception to June 2025: PubMed, Embase, CINAHL, Cochrane Library, Web of Science, PsycINFO, CNKI, and VIP. Additional grey literature sources were screened, including ClinicalTrials.gov and ProQuest Dissertations and Theses. For ProQuest, the search was restricted to the most recent three years to minimize duplication of evidence already published as journal articles. Search strategies combined Medical Subject Headings (MeSH) and free-text terms, tailored to each database. The complete search strategies are provided in Appendix 2.

### 2.2 Eligibility criteria

Eligible studies were randomized controlled trials enrolling older adults (≥60 years) diagnosed with mild cognitive impairment using internationally recognized criteria. Interventions included any non-pharmacological nutritional supplement, administered alone or in combination, compared with placebo or usual care. Studies were required to report global cognitive outcomes measured by validated tools (e.g., MMSE, MoCA, ADAS-Cog), while secondary outcomes included peripheral biomarkers such as BDNF, Aβ42, and Aβ40. Trials involving pharmacological agents, exercise, or cognitive training; participants with dementia or other neurological disorders; non-randomized designs; and studies with insufficient outcome data or no full text were excluded.

### 2.3 Study selection and data extraction

All records were imported into EndNote 20 for duplicate removal, after which two reviewers independently screened titles and abstracts, evaluated full texts, and extracted data. Discrepancies were resolved through discussion or consultation with a third reviewer. Extracted data included study characteristics, participant demographics, diagnostic criteria, intervention details, comparator conditions, and outcome statistics (means, standard deviations, and sample sizes).

### 2.4 Risk of bias assessment

The methodological quality of all included randomized controlled trials was evaluated using the Cochrane Risk of Bias Tool (RoB 1.0) as described in the Cochrane Handbook for Systematic Reviews of Interventions (17). The tool assesses seven domains: random sequence generation, allocation concealment, blinding of participants and personnel, blinding of outcome assessment, completeness of outcome data, selective reporting, and other sources of bias. Each domain was rated as low, high, or unclear risk of bias. Disagreement between reviewers was resolved through discussion or adjudication by a third reviewer. Based on the number and importance of domains rated as low risk, studies were categorized as grade A (low risk), grade B (moderate risk), or grade C (high risk). Studies rated as grade C were excluded from quantitative synthesis. The quality assessment results are summarized in Appendix 3 (Supplementary Figure S1).

### 2.5 Statistical analysis

All statistical analyses were conducted using RevMan 5.4 (for pairwise meta-analyses and forest plots) and R version 4.2.0, with the netmeta package applied for the frequentist network meta-analysis (NMA). For each included study, we extracted post-intervention means, standard deviations, and sample sizes for both intervention and control groups. For continuous cognitive outcomes, mean difference (MD) with 95% confidence intervals (CI) was calculated when studies used the same measurement scale. When heterogeneous cognitive scales were used, standardized mean difference (SMD) with 95% *CI* was applied to account for between-scale variability. Statistical heterogeneity was quantified using the I^2^ statistic; a random-effects model was implemented when *I^2^* exceeded 50%, whereas a fixed-effect model was used otherwise.

Pre-specified subgroup analyses were performed according to supplement categories (vitamins, omega-3 polyunsaturated fatty acids, probiotics, or plant-derived supplements) and physiological biomarkers (BDNF, Aβ42, Aβ40). Supplement categories were defined based on the primary active components reported in the original studies. Specifically, vitamins included B vitamins (e.g., folic acid) and vitamin D; polyunsaturated fatty acids (PUFAs) included eicosapentaenoic acid (EPA), docosahexaenoic acid (DHA), or their combination; probiotics referred to single- or multi-strain live bacterial preparations; and plant-derived supplements included extracts such as *Ginkgo biloba* or Spirulina.

For the network meta-analysis, a connected evidence network was constructed using placebo as the common comparator. Relative treatment effects were estimated under a frequentist graph-theoretical framework. Ranking probabilities for each intervention were quantified using P-scores, which reflect the surface under the cumulative ranking curve. Local inconsistency was evaluated using node-splitting methods, and global inconsistency was assessed by comparing random-effects variance across the network.

To evaluate robustness of findings, several sensitivity analyses were conducted: (1) exclusion of studies judged to have high risk of bias; and (2) restriction to studies using the same cognitive assessment instrument (MMSE, MoCA, or FSIQ) as the primary endpoint.

## 3 Results

**Figure 1:**
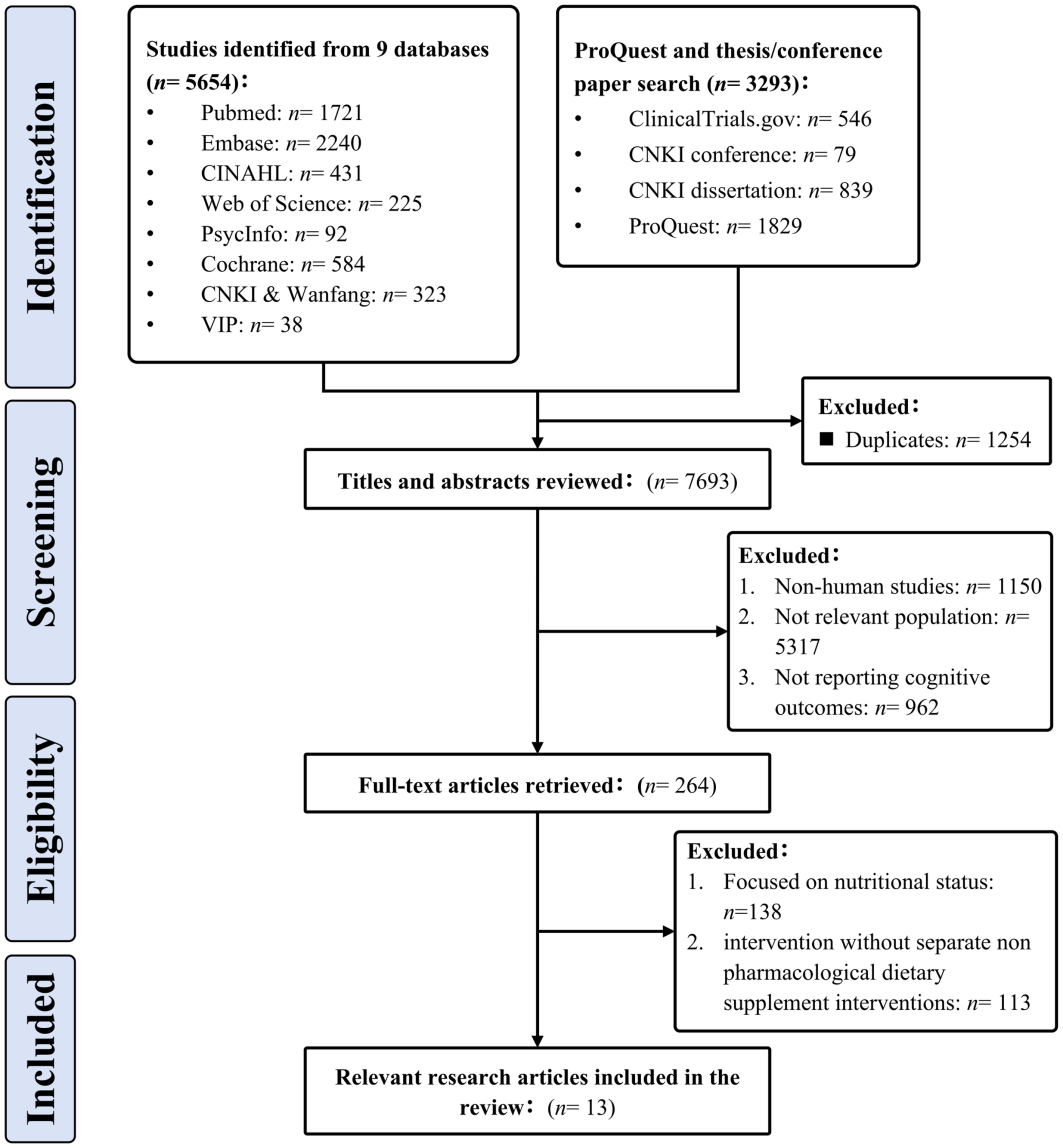
Flow chart of literature screening.

A total of 8,947 records were initially identified through the database search. After removing 1,254 duplicates, 7,693 records underwent title and abstract screening. Of these, 264 articles were retrieved for full-text assessment. A total of 138 studies were excluded because they focused on nutritional status rather than supplementation, and 113 studies were excluded because the intervention was not an isolated non-pharmacological dietary supplement. Ultimately, 13 randomized controlled trials met the inclusion criteria, comprising eight Chinese and five English publications. All reviewers reached full agreement on study selection and quality assessment. The flow of literature screening is presented in Figure 1.

### 3.1 Study and patient characteristics

This study included 13 randomized controlled trials involving a total of 2,451 participants. Most participants (46.88%) were recruited from China. The publication years of the included studies spanned from 2008 to 2023. Geographically, the studies were conducted in China (18–25), France and Monaco (26), South Korea (27), Japan (28), the Netherlands (29), and the Taiwan region of China (20). The interventions evaluated were diverse in form, including folic acid, docosahexaenoic acid (DHA), eicosapentaenoic acid (EPA), multi-strain probiotics, vitamin D, and *Ginkgo biloba* leaf extract, among others. The duration of these interventions ranged from 12 weeks to 3 years. Control groups predominantly received usual care, placebo, or basic standard treatment. Detailed characteristics of the included studies are provided in Appendix 3 (Supplementary Table S1).

### 3.2 Risk of Bias assessment

Four studies (19, 27, 28, 30) were rated as Grade A (low risk of bias), eight (18, 20, 21, 23–26, 29) as Grade B (moderate risk), and one (22) as Grade C (high risk), indicating that the majority of studies were considered to have low-to-moderate risk of bias. However, two studies (21, 22) were judged to be at high risk of bias in domains including allocation concealment, blinding of participants, and blinding of personnel due to unclear reporting of participant blinding, lack of information on assessor blinding, and inadequate description of allocation sequence generation. A detailed summary of the risk of bias assessment is provided in Appendix 3 (Supplementary Figure S1).

### 3.3 Meta-analysis findings

#### 3.3.1 Primary outcome - cognitive function

**Figure 2:**
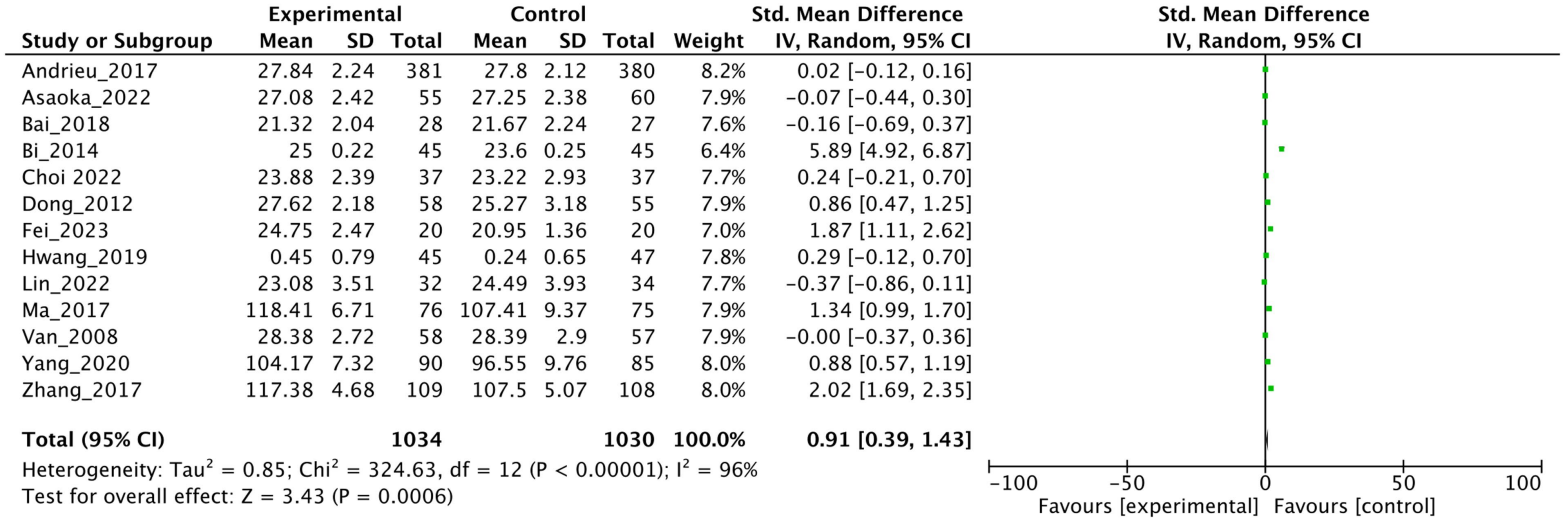
Forest plot of dietary supplements compared with control for global cognitive function.

13 studies reported the effects of dietary supplements on global cognitive function. A random-effects model was used in the conventional meta-analysis. As presented in Figure 2. The results demonstrated that dietary supplement interventions significantly improved cognitive function in patients with mild cognitive impairment (*SMD* = 0.91, 95%*CI*: 0.39 ~ 1.53, *p* < 0.001) compared with the control group. However, substantial heterogeneity was observed among the included studies (*I*^2^ = 96%).

**Figure 3:**
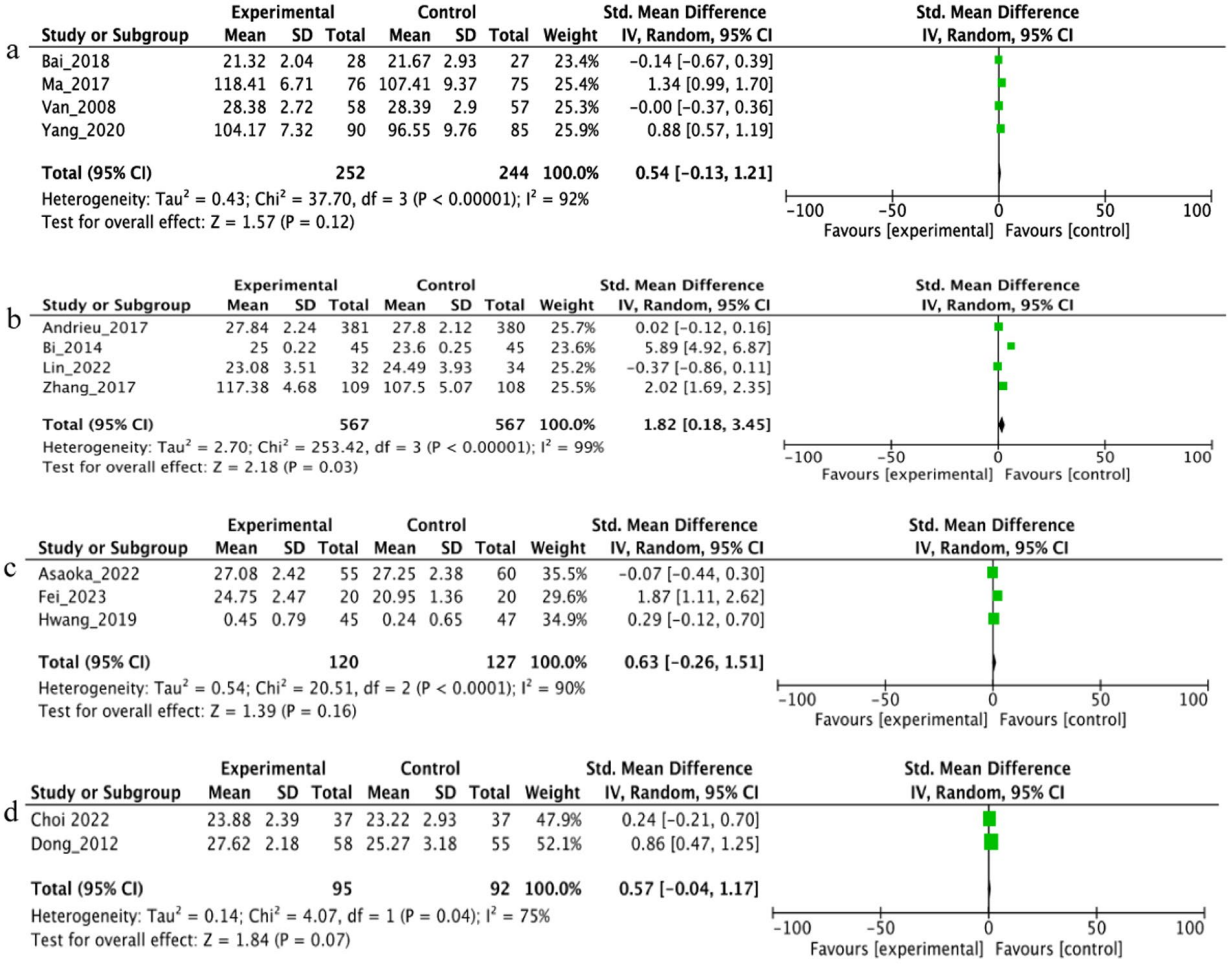
Subgroup analysis of the effects of dietary supplements on cognitive function: **(a)** Vitamins, **(b)** polyunsaturated fatty acids (PUFAs), **(c)** probiotics, **(d)** plant-based supplements.

To explore potential sources of heterogeneity, we conducted a subgroup meta-analysis by categorizing the 13 included studies into four distinct supplement types: vitamins, polyunsaturated fatty acids (PUFAs), probiotics, and plant-based supplements. The analysis revealed that PUFAs [4 studies: (19, 20, 22, 26)] were associated with a statistically significant improvement in cognitive function, with a pooled *SMD* of 1.82 (95% *CI*: 0.18 to 3.45, *p* = 0.03). However, considerable heterogeneity was observed within this subgroup (*I^2^* = 99%). In contrast, no significant effects were observed for the other supplement categories: Vitamin supplements [4 studies: (18, 24, 25, 29)]: pooled *SMD* = 0.54 (95% *CI*: −0.13 to 1.21, *p* = 0.12, *I^2^* = 92%); Probiotic supplements [3 studies: (21, 27, 28)]: pooled *SMD* = 0.63 (95% *CI*: −0.26 to 1.51, *p* = 0.16, *I^2^* = 90%); Plant-based supplements [2 studies: (23, 30)]: pooled *SMD* = 0.57 (95% *CI*: −0.04 to 1.17, *p* = 0.07, *I^2^* = 75%). Forest plots for all subgroup analyses are presented in Figures 3a–d.

#### 3.3.2 Secondary outcomes—physiological biomarkers

A subgroup meta-analysis was performed on data from six studies, categorized by physiological biomarkers: BDNF, Aβ42, and Aβ40 (18–21, 27, 30). The pooled analysis of four studies on BDNF showed low heterogeneity; however, there was no statistically significant difference in BDNF levels between the intervention and control groups (*SMD* = 0.20, 95% *CI*: −0.03 to 0.44, *p* = 0.09).

In the analysis of Aβ42 [3 studies: (18, 19, 30)], the intervention group showed significantly lower levels than the control group (*SMD* = −1.81, 95% *CI*: −3.55 to −0.06, *p* = 0.04), though substantial heterogeneity was present, warranting cautious interpretation of this result. For Aβ40 [3 studies: (18, 19, 30)], supplementation was associated with a significant reduction in levels (*SMD* = −0.45, 95% *CI*: −0.76 to −0.13, *p* = 0.005), with moderate heterogeneity observed (*I*^2^ = 60%), suggesting relative robustness of this finding. The corresponding forest plots are provided in Appendix 3 (Supplementary Figure S2).

### 3.4 Network meta-analysis

**Figure 4:**
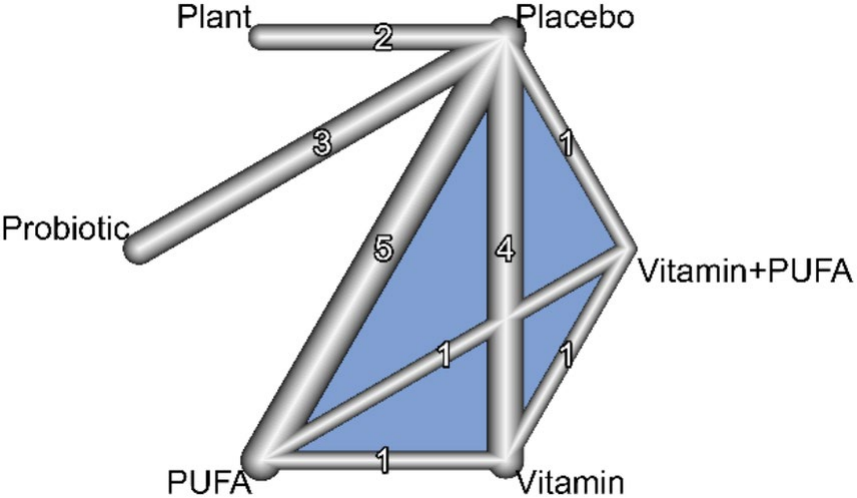
Network of interventions for cognitive outcomes.

The network meta-analysis for cognitive outcomes included 13 studies investigating five types of interventions. The structure of the evidence network is shown in Figure 4. The PUFA group had the largest sample size and thus the largest node (*n* = 5), followed by the Vitamin, Probiotic, and Plant groups, while only one study investigated the Vitamin + PUFA combination. Each nutritional intervention was supported by at least one direct comparison with the control group. The overall network was well-connected, though studies using probiotics as an intervention appeared relatively isolated, with no subnetworks linked to them. A closed loop was formed among PUFA, Vitamin, and Vitamin + PUFA, indicating the presence of direct evidence between these three interventions. Overall, the network met the basic assumptions for conducting NMA.

**Figure 5:**
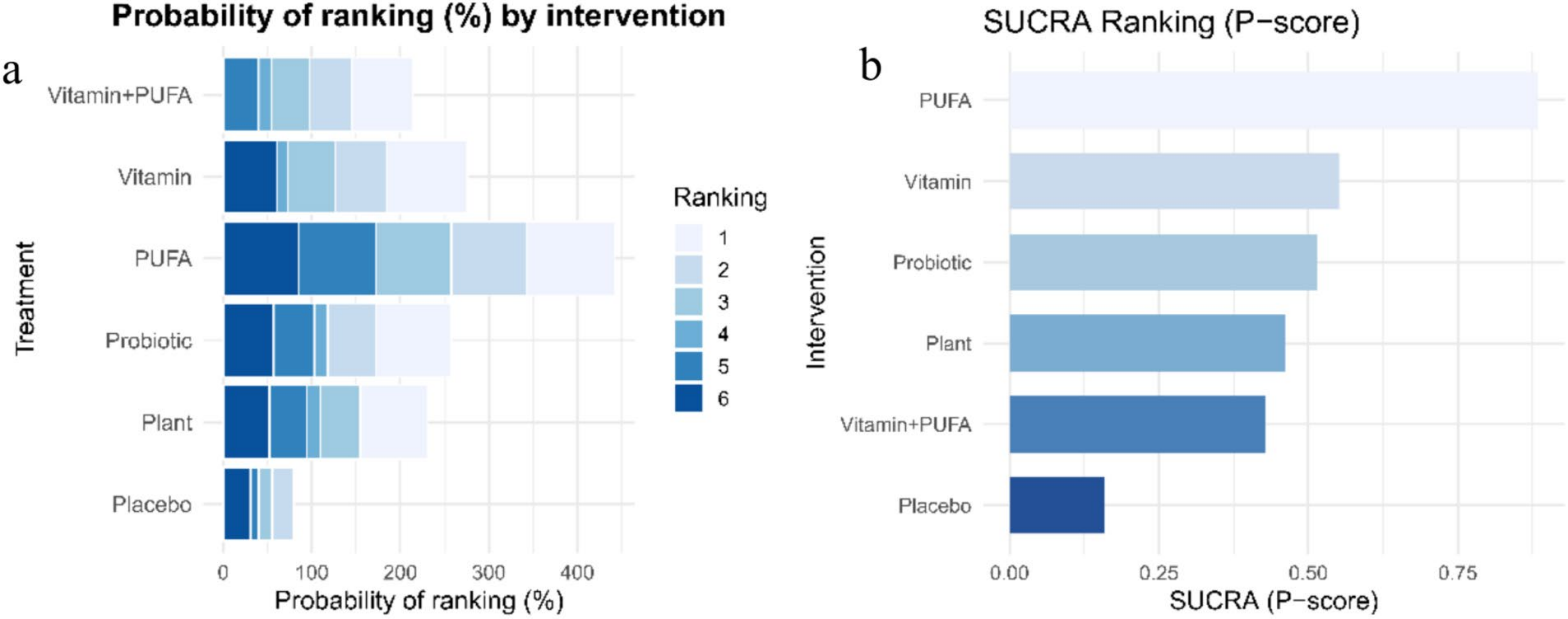
Ranking of interventions for cognitive improvement. **(a)** Rank probability distribution, showing the probability of each intervention being the best, second best, and so on. **(b)** SUCRA values, indicating the overall efficacy of each intervention, with higher values representing better performance (SUCRA: surface under the cumulative ranking curve).

**Figure 6:**
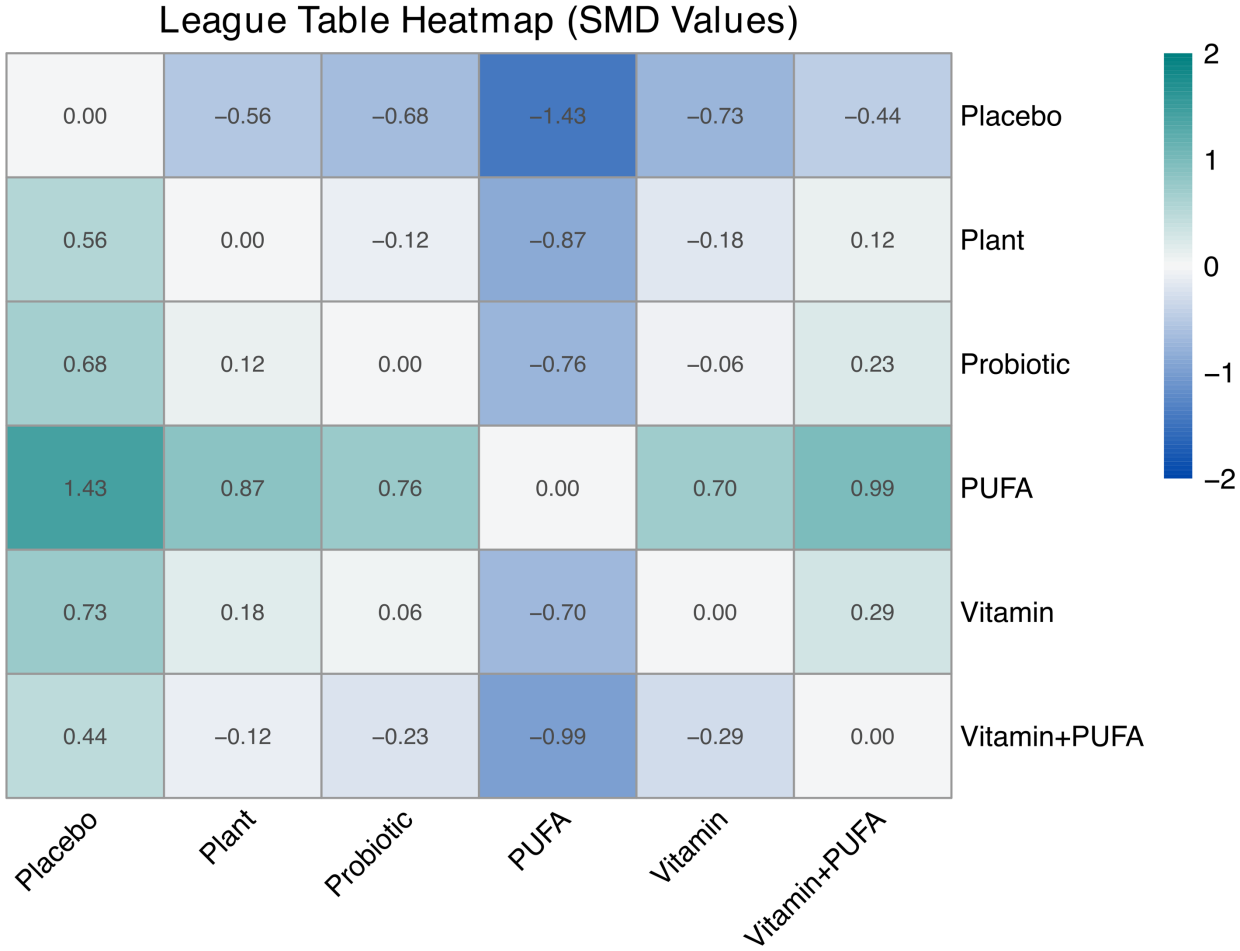
League table of relative effect sizes for cognitive function between intervention and control.

Figure 5 presents the ranking probability distributions and cumulative ranking curves (SUCRA values) of different interventions for cognitive improvement. In Figure 5a, the color intensity represents the probability of each intervention achieving a specific rank, with PUFA showing the highest probability of being the most effective (ranked first). Figure 5b displays the surface under the cumulative ranking curve (SUCRA), indicating that PUFA had the highest SUCRA value (86.62%). As shown in Figure 6, all comparisons between PUFA and other interventions yielded positive SMD values, consistently supporting PUFA as the most effective intervention for improving cognitive function. Vitamin ranked second (SUCRA, 55.62%). In contrast, the combination of Vitamin and PUFA showed the lowest probability of cognitive improvement (SUCRA, 42.07%), with non-significant effect sizes compared to PUFA and Vitamin alone.

### 3.5 Network meta-analysis of polyunsaturated fatty acid subtypes

To further investigate the contribution of specific types of polyunsaturated fatty acid (PUFA) supplements to the overall effect, a subgroup network meta-analysis was conducted by categorizing interventions into DHA, EPA, and DHA + EPA. The network included four studies (19, 20, 22, 26), encompassing four interventions: DHA, EPA, DHA + EPA combination, and placebo. Among these, Lin_2022 (20) was a multi-arm trial that provided direct comparisons between all three supplement interventions and the control group. The results indicated that the DHA + EPA combination was most likely to be the optimal intervention (P-score = 0.81), followed by DHA alone (P-score = 0.58), and EPA alone (P-score = 0.48). The SUCRA value for DHA + EPA was significantly higher than those for DHA and EPA, indicating its superior cumulative ranking. The rank probability distribution, league table, and cumulative ranking plot are presented in Appendix 3 (Supplementary Figures S4, S5).

### 3.6 Sensitivity analysis

To assess the robustness of the primary analysis, sensitivity analyses were performed from two perspectives: exclusion of low-quality studies and restriction to studies using a specific assessment instrument. (1) Sensitivity Analysis by Excluding Low-Quality Studies. After removing two studies rated as having a ‘high risk of bias’ (21, 22), the pooled effect size based on the remaining 11 higher-quality studies decreased but remained statistically significant (pooled effect size = 0.47, 95%*CI*: 0.03 to 0.90, *p* = 0.04). Heterogeneity did not change substantially. These results suggest that while low-quality studies had some influence on the overall effect magnitude, the direction and significance of the primary findings were not altered, supporting the robustness of the main results. (2) Sensitivity Analysis Restricted to Studies Using the MMSE. When the analysis was limited to the nine studies that used the MMSE as the outcome measure, a significant benefit was observed in the intervention group compared to the control group (*SMD* = 0.80, 95%*CI*: 0.19 to 1.41; *p* = 0.01). Despite high heterogeneity (*I^2^* = 96%, *p* < 0.00001), the results consistently supported the superiority of the intervention group on MMSE outcomes. Detailed forest plots are provided in Appendix 3 (Supplementary Figures S6, S7).

## 4 Discussion

This systematic review and network meta-analysis synthesized evidence from 13 randomized controlled trials involving 2,451 older adults with mild cognitive impairment and evaluated the comparative effectiveness of multiple non-pharmacological dietary supplements on cognitive outcomes and physiological biomarkers. Overall, dietary supplementation was associated with a modest improvement in global cognition relative to control groups, although substantial heterogeneity was observed across studies. Among all interventions, polyunsaturated fatty acids - particularly formulations containing DHA and EPA - tended to rank highest in both pairwise and network meta-analyses, achieving the highest SUCRA values. Supplementation also resulted in a significant reduction in circulating Aβ42 levels, whereas effects on BDNF and Aβ40 were not statistically significant. Sensitivity analyses demonstrated attenuation of effect sizes after exclusion of studies with high risk of bias, but the overall direction of findings remained unchanged, indicating reasonable robustness while warranting cautious interpretation. Importantly, although statistically significant improvements in global cognition were observed, these findings do not necessarily translate into clinically meaningful benefits. The magnitude of the pooled effect sizes was modest, and it remains unclear whether such changes correspond to perceptible improvements in daily functioning or delay in disease progression in individuals with MCI. This distinction is particularly important given that most included trials had relatively short intervention durations, which may be insufficient to capture sustained or clinically relevant cognitive changes.

Our findings strengthen accumulating evidence supporting PUFA supplementation - especially combined DHA and EPA - as a promising strategy for cognitive preservation in MCI. This aligns with several well-designed clinical trials. Lin et al. demonstrated that DHA + EPA supplementation significantly improved ADAS - Cog scores compared with placebo (20), while Zhang et al. reported long-term cognitive benefits associated with DHA alone (19). The ranking patterns observed in the network meta-analysis, with combined DHA + EPA outperforming single supplements, are biologically plausible. DHA is essential for neuronal membrane fluidity and synaptic function (31), whereas EPA confers anti-inflammatory and neuroprotective effects (32); their combination may exert synergistic actions by simultaneously modulating membrane dynamics, synaptic plasticity, and inflammatory pathways (33).

The observed reduction in Aβ42 levels further supports mechanisms identified in preclinical research, where *ω*-3 fatty acids enhance non-amyloidogenic APP processing and attenuate Aβ deposition (34). By contrast, vitamin-based supplements yielded no statistically significant cognitive improvement—a finding consistent with RCTs that reported null effects of high-dose B vitamins in unselected MCI populations (35, 36). Variability in baseline nutritional status, wide differences in dosage (e.g., 0.4 mg vs. 5 mg folate), and the absence of hyperhomocysteinemia in many participants may partly explain these discrepancies (18). Evidence for probiotics and plant-derived supplements remains limited (37), predominantly due to small sample sizes and inconsistent methodological quality (38).

The superior performance of PUFA supplements may be explained by their established neurobiological roles in maintaining neuronal membrane integrity (39), modulating synaptic function, and exerting anti-inflammatory effects (40). The significant reduction in Aβ42 observed in this review further supports their involvement in modulating amyloidogenic processes.

Conversely, the lack of measurable improvement in BDNF levels suggests several possibilities: peripheral BDNF may not accurately reflect central neurotrophic activity; intervention durations in included studies may be insufficient to elicit sustained increases; or the effective threshold dose required for neurotrophic modulation was not reached. These findings underscore the need for biomarker-rich trials incorporating central and peripheral measures. Moreover, recent research suggests that composite or ratio-based indicators may provide greater diagnostic and prognostic value than single analytes. In particular, the Aβ42/Aβ40 ratio has been shown to outperform individual Aβ measures in reflecting amyloid pathology and disease progression. In addition, plasma biomarkers such as phosphorylated tau (e.g., p-tau181 and p-tau217) and glial fibrillary acidic protein (GFAP) have demonstrated strong potential for tracking neurodegeneration and evaluating treatment response (41).

The substantial heterogeneity observed (*I*^2^ = 96%) substantially limits the strength and certainty of the conclusions. First, supplementation protocols differed markedly in dose, duration, and composition, particularly within PUFA trials where DHA/EPA ratios varied widely. The superior ranking of combined DHA + EPA suggests that compositional differences may have contributed significantly to variability. Second, the methodological quality of included trials varied, studies with unclear allocation concealment or insufficient blinding likely inflated effect estimates. Indeed, removal of high-risk studies reduced the pooled effect from *SMD* = 0.91 to *SMD* = 0.47. Third, participant characteristics differed across trials, including age ranges, diagnostic criteria, baseline cognitive levels, and comorbidities. Some trials focused on specific subgroups—such as individuals with hypercholesterolemia—potentially enhancing the apparent efficacy of PUFAs (42). Finally, heterogeneity in cognitive assessment tools (MMSE, MoCA, ADAS-Cog, WAIS-RC) may also have influenced results; while SMD standardization mitigates scale differences, variable sensitivity and ceiling effects - especially with MMSE—may still introduce measurement variability (43).

Several limitations should be acknowledged. First, the overall methodological quality of included studies was moderate, and two trials demonstrated high risk of bias due to inadequate blinding and unclear allocation concealment, which may overestimate treatment effects. Second, sample sizes in several trials were small, reducing statistical power and precision. The predominance of studies conducted in China may limit generalizability to populations with different dietary profiles, genetic backgrounds, or nutritional statuses. Third, although comprehensive searches were performed, the relatively small number of eligible studies and limited inclusion of grey literature prevented formal assessment of publication bias. Finally, most included interventions were of relatively short duration (typically less than 12 months), which limits the ability to determine whether the observed cognitive improvements are sustained over time or translate into clinically meaningful outcomes, such as delayed progression to dementia.

## 5 Conclusion

This systematic review and network meta-analysis provides comparative evidence on the effectiveness of dietary supplements for cognitive enhancement in MCI. PUFAs, particularly DHA + EPA formulations, showed the most favorable ranking in this analysis. However, the substantial heterogeneity and variability in study quality limit the strength of this conclusion. Future well-designed, adequately powered RCTs with extended follow-up, biomarker-enriched endpoints, and clearly defined subgroups (e.g., APOE-ε4 carriers, nutritional deficiency profiles) are essential to determine optimal formulations, target populations, and the potential role of these supplements as adjunct strategies in clinical management of MCI. Incorporating cost-effectiveness and real-world implementation analyses will further support evidence-based use of dietary supplementation in MCI management.

## Data Availability

The original contributions presented in the study are included in the article/Supplementary material, further inquiries can be directed to the corresponding author/s.
