# Supplementary material for: Effects of dietary supplements on cognitive outcomes and physiological biomarkers in mild cognitive impairment: a systematic review and network meta-analysis
Source: Front Nutr. 2026 Apr 29;13:1775177. doi: 10.3389/fnut.2026.1775177 (PMC13167511; doi:10.3389/fnut.2026.1775177)
Supplement: Supplementary file 1 [file Data_Sheet_1.PDF]

Table S1 characteristics of studies

No.	Included Study	Country	Design	Sample Size	Study Population	Intervention	Control Group	Evaluation Tool
1	Ma_2017	China	RCT	180 MCI patients (Intervention n=90, Control n=90)	≥65 years, MCI	Folic acid: 400μg/day, 24 months	Routine treatment	Wechsler Adult Intelligence Scale (WAIS-RC)
2	Zhang_2017	China	RCT	240 MCI patients (DHA n=120, Placebo n=120)	≥65 years, MCI	DHA: 2g/day, 24 months	Corn oil	Wechsler Adult Intelligence Scale (WAIS-RC)
3	Andrieu_2017	France and Monaco	RCT	1680 elderly with memory complaints	≥70 years, memory complaints, non-demented community-dwelling elderly	Omega-3: 800mg DHA + 225mg EPA/day, 3 years (extracted data)	Placebo	Composite Cognitive Z-score
4	Lin_2022	Taiwan, China	RCT	163 MCI (DHA n=41, EPA n=40, Combined n=42, Placebo n=40)	65-94 years, MCI	Omega-3: 1. DHA (0.7g/day), 2. EPA (1.6g/day), 3. EPA+DHA (24 months)	Soybean oil	Cognitive Function (ADAS-Cog, MMSE)
5	Hwang_2019	Korea	RCT	100 MCI patients (Intervention n=50, Placebo n=50)	55-85 years, MCI	Lactobacillus plantarum C29 fermented soybean (DW2009): 800mg/day, 12 weeks	Placebo (cellulose)	Composite Cognitive Function Score (CNT)
6	Asaoka_2022	Japan	RCT	130 MCI (115 analyzed)	65-88 years, MCI	Bifidobacterium breve MCC1274: 2×10^{10} CFU/day, 24 weeks	Placebo	Cognitive Function (ADAS-Jcog, MMSE)
7	Fei_2023	China	RCT	42 MCI patients (Probiotics n=21, Placebo n=21)	>60 years, MCI	Compound probiotics: 2g/day, 12 weeks	Placebo (starch)	Cognitive Function (MMSE, MoCA), Sleep Quality (PSQI)
8	Choi_2022	Korea	RCT	80 MCI volunteers	>60 years, MCI	Arthrospira maxima extract (SM70EE): 1g/day, 12 weeks	Placebo	Visual Learning, Visual Working Memory (CNT)
9	Bi_2014	China	RCT	90 MCI patients with hypercholesterolemia (Intervention n=45, Control n=45)	>60 years, MCI	Fish oil capsules (1000mg) + dietary intervention, 12 weeks	Dietary intervention only	Cognitive Ability (MoCA)

No.	Included Study	Country	Design	Sample Size	Study Population	Intervention	Control Group	Evaluation Tool
10	Dong_2012	China	RCT	113 MCI patients (Treatment n=58, Control n=55)	60-85 years, MCI	Ginkgo biloba + basic treatment, 1 year	Basic treatment (including exercise guidance)	Cognitive Function (MMSE, CDT), Dementia Conversion Rate
11	Yang_2020	China	RCT	183 MCI patients (Intervention n=93, Placebo n=90)	≥65 years, MCI	Vitamin D: 800 IU/day, 12 months	Placebo (starch granules)	Wechsler Adult Intelligence Scale (WAIS-RC)
12	Van_2008	Netherlands	RCT	152 MCI patients randomized to two interventions	70-80 years, MCI	Supplement intervention: 5mg folic acid, 0.4mg B12, 50mg B6/day, 1 year	Placebo pill	Cognitive Function (neuropsychological tests)
13	Bai_2018	China	RCT	138 MCI patients (Placebo n=33, Folic acid n=35, DHA n=36, Combined n=34)	≥60 years, MCI	1. Folic acid: 800µg/day, 2. DHA: 800mg/day, 3. Combined: Folic acid 800µg/day + DHA 800mg/day (6 months)	Corn starch + corn oil	Wechsler Adult Intelligence Scale (WAIS-RC)

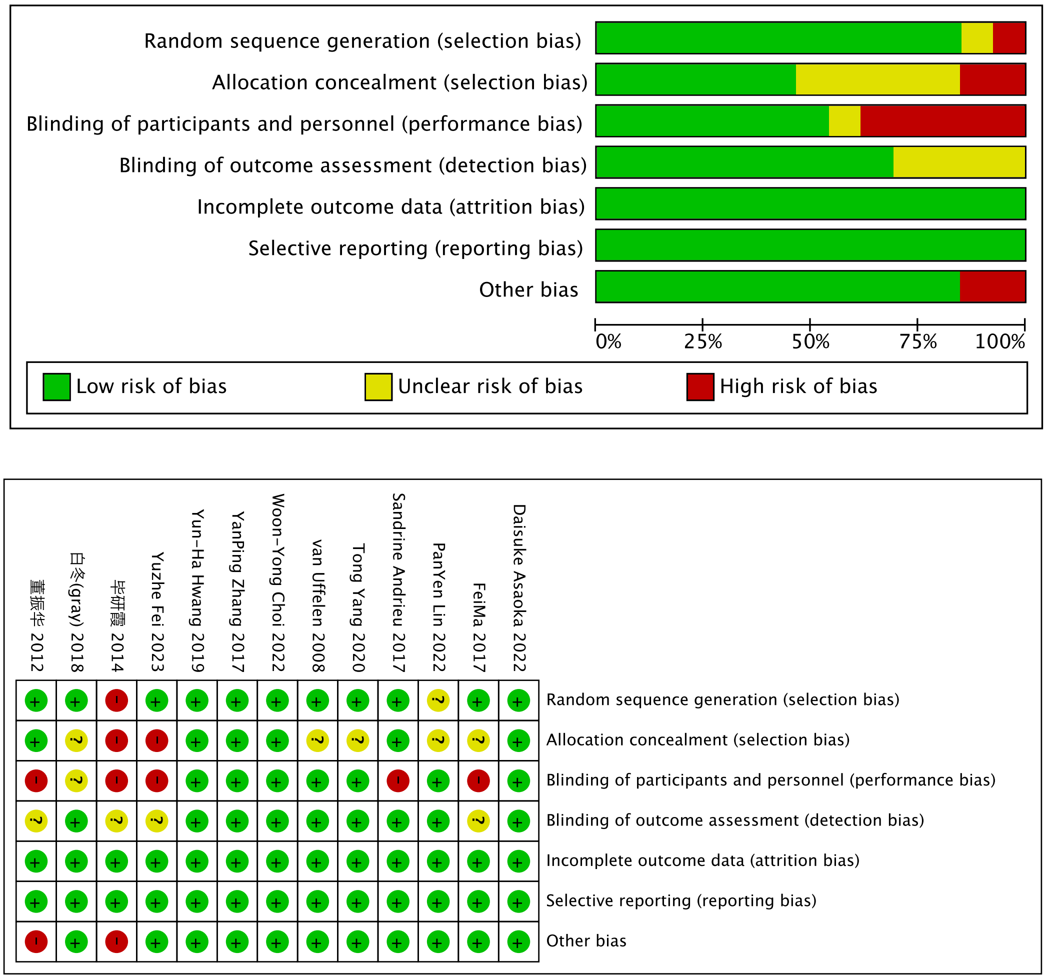

Fig S1. Summary plot of study quality assessment.

a

Study or Subgroup	Experimental Mean	Experimental SD	Experimental Total	Control Mean	Control SD	Control Total	Weight	Std. Mean Difference IV, Random, 95% CI
Choi 2022	−2,236.5	3,359.17	37	−3,500.73	3,319.6	37	26.6%	0.37 [−0.09, 0.83]
Fei_2023	0.33	1.22	20	0.03	1.45	20	14.6%	0.22 [−0.40, 0.84]
Hwang_2019	412.7	7,212.4	45	−1,034.3	5,644.5	47	33.5%	0.22 [−0.19, 0.63]
Lin_2022	−87.71	130.61	35	−85.71	130.6	34	25.3%	−0.02 [−0.49, 0.46]
**Total (95% CI)**			**137**			**138**	**100.0%**	**0.20 [−0.03, 0.44]**

Heterogeneity: $Tau^2 = 0.00$; $Chi^2 = 1.37$, df = 3 (P = 0.71); $I^2 = 0\%$
Test for overall effect: Z = 1.67 (P = 0.09)

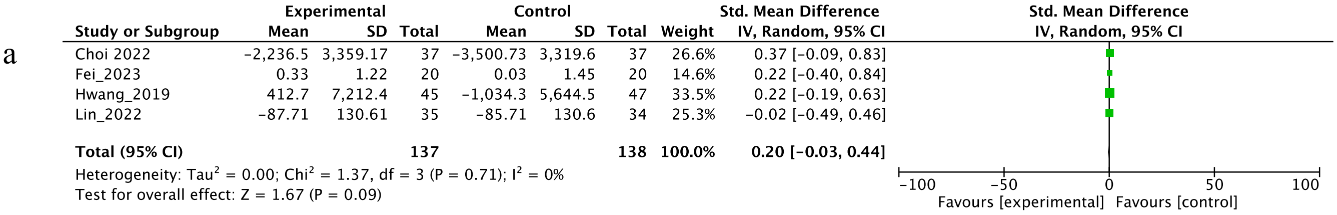

b

Study or Subgroup	Experimental Mean	Experimental SD	Experimental Total	Control Mean	Control SD	Control Total	Weight	Std. Mean Difference IV, Random, 95% CI
Choi 2022	−0.27	1.15	37	−0.04	1.17	37	33.2%	−0.20 [−0.65, 0.26]
Ma_2017	−2.84	0.93	76	−1.1	1.01	75	33.5%	−1.78 [−2.16, −1.41]
Zhang_2017	−2.84	0.98	109	−0.1	0.55	108	33.3%	−3.43 [−3.85, −3.01]
**Total (95% CI)**			**222**			**220**	**100.0%**	**−1.81 [−3.55, −0.06]**

Heterogeneity: $Tau^2 = 2.33$; $Chi^2 = 104.71$, df = 2 (P < 0.00001); $I^2 = 98\%$
Test for overall effect: Z = 2.03 (P = 0.04)

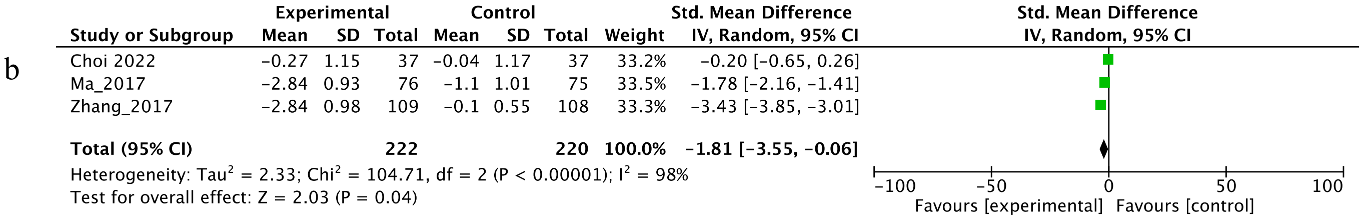

c

Study or Subgroup	Experimental Mean	Experimental SD	Experimental Total	Control Mean	Control SD	Control Total	Weight	Std. Mean Difference IV, Random, 95% CI
Choi 2022	4.32	25.15	37	6.28	24.88	37	25.7%	−0.08 [−0.53, 0.38]
Ma_2017	−0.62	0.92	76	−0.1	1.31	75	35.1%	−0.46 [−0.78, −0.13]
Zhang_2017	−0.63	0.83	109	−0.07	0.81	108	39.2%	−0.68 [−0.95, −0.41]
**Total (95% CI)**			**222**			**220**	**100.0%**	**−0.45 [−0.76, −0.13]**

Heterogeneity: $Tau^2 = 0.05$; $Chi^2 = 5.03$, df = 2 (P = 0.08); $I^2 = 60\%$
Test for overall effect: Z = 2.79 (P = 0.005)

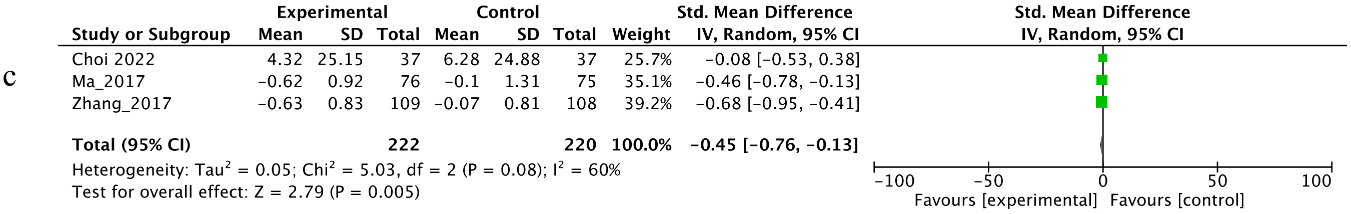

Fig. S2 Subgroup analyses of physiological biomarkers: (a) BDNF; (b) Aβ42; (c) Aβ40.

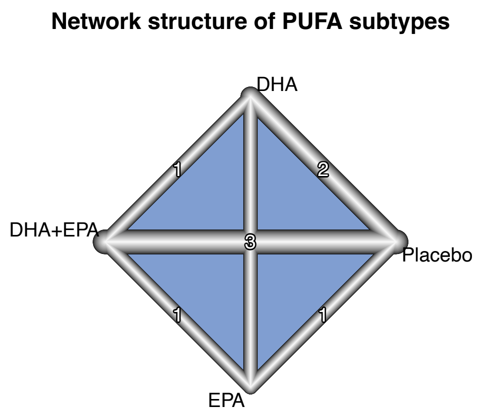

Fig. S3 Network evidence map of the included interventions.

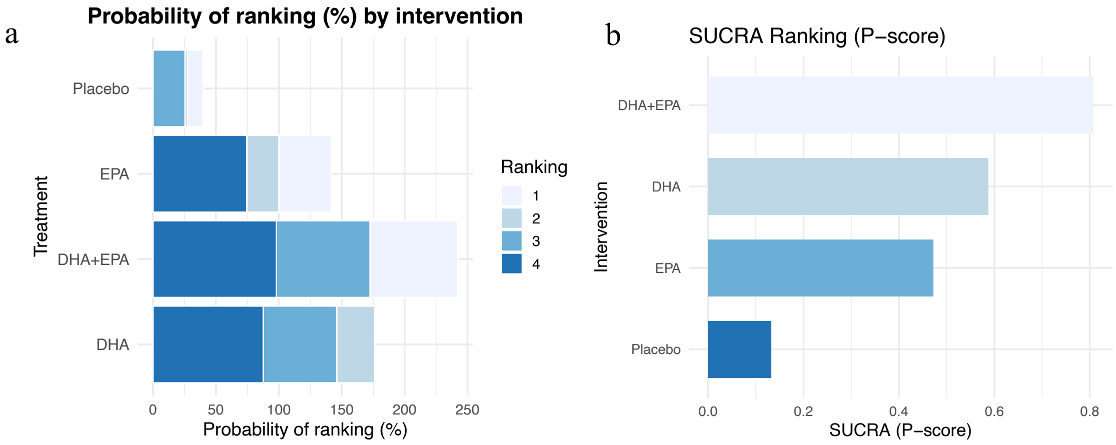

Fig. S4 Ranking analyses of the cognitive improvement effects of the interventions. (a) Ranking probability distribution showing the probability of each intervention occupying each possible rank. (b) SUCRA-based ranking, presented as the surface under the cumulative ranking curve.

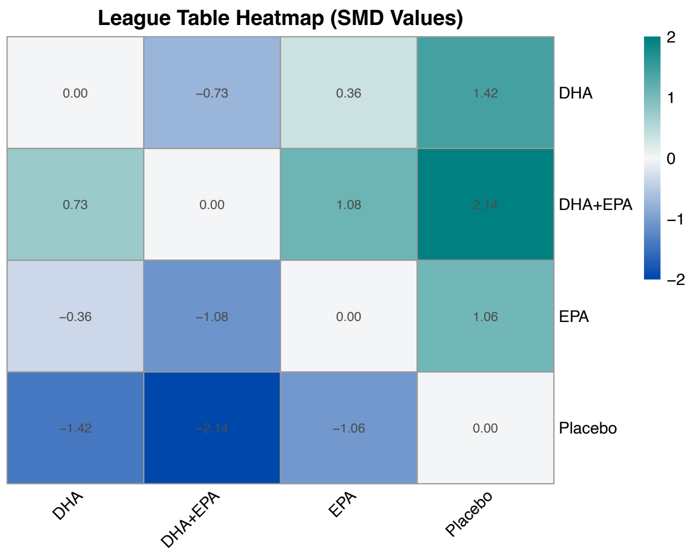

Fig. S5 League table heatmap of pairwise treatment comparisons

Study or Subgroup	Experimental Mean	SD	Total	Control Mean	SD	Total	Weight	Std. Mean Difference IV, Random, 95% CI
Andrieu_2017	27.84	2.24	381	27.8	2.12	380	9.7%	0.02 [−0.12, 0.16]
Asaoka_2022	27.08	2.42	55	27.25	2.38	60	9.2%	−0.07 [−0.44, 0.30]
Bai_2018	21.32	2.04	28	21.67	2.24	27	8.6%	−0.16 [−0.69, 0.37]
Choi 2022	23.88	2.39	37	23.22	2.93	37	8.8%	0.24 [−0.21, 0.70]
Dong_2012	27.62	2.18	58	25.27	3.18	55	9.1%	0.86 [0.47, 1.25]
Hwang_2019	0.45	0.79	45	0.24	0.65	47	9.0%	0.29 [−0.12, 0.70]
Lin_2022	23.08	3.51	32	24.49	3.93	34	8.7%	−0.37 [−0.86, 0.11]
Ma_2017	118.41	6.71	76	107.41	9.37	75	9.2%	1.34 [0.99, 1.70]
Van_2008	28.38	2.72	58	28.39	2.9	57	9.2%	−0.00 [−0.37, 0.36]
Yang_2020	104.17	7.32	90	96.55	9.76	85	9.3%	0.88 [0.57, 1.19]
Zhang_2017	117.38	4.68	109	107.5	5.07	108	9.3%	2.02 [1.69, 2.35]
**Total (95% CI)**			**969**			**965**	**100.0%**	**0.47 [0.03, 0.90]**

Heterogeneity: $Tau^2 = 0.51$; $Chi^2 = 189.02$, df = 10 ($P < 0.00001$); $I^2 = 95\%$
Test for overall effect: Z = 2.10 (P = 0.04)

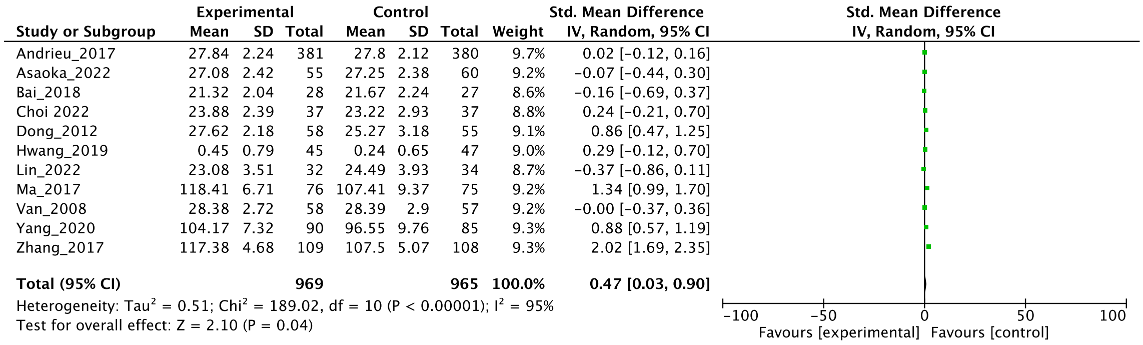

Fig. S6 Forest plot of the pooled effect after excluding low-quality studies.

Study or Subgroup	Experimental Mean	SD	Total	Control Mean	SD	Total	Weight	Std. Mean Difference IV, Random, 95% CI
Andrieu_2017	27.84	2.24	381	27.8	2.12	380	12.0%	0.02 [−0.12, 0.16]
Asaoka_2022	27.08	2.42	55	27.25	2.38	60	11.6%	−0.07 [−0.44, 0.30]
Bai_2018	21.32	2.04	28	21.67	2.24	27	11.1%	−0.16 [−0.69, 0.37]
Bi_2014	25	0.22	45	23.6	0.25	45	9.3%	5.89 [4.92, 6.87]
Choi 2022	23.88	2.39	37	23.22	2.93	37	11.3%	0.24 [−0.21, 0.70]
Dong_2012	27.62	2.18	58	25.27	3.18	55	11.6%	0.86 [0.47, 1.25]
Fei_2023	24.75	2.47	20	20.95	1.36	20	10.2%	1.87 [1.11, 2.62]
Lin_2022	23.08	3.51	32	24.49	3.93	34	11.2%	−0.37 [−0.86, 0.11]
Van_2008	28.38	2.72	58	28.39	2.9	57	11.6%	−0.00 [−0.37, 0.36]
**Total (95% CI)**			**714**			**715**	**100.0%**	**0.80 [0.19, 1.41]**

Heterogeneity: $Tau^2 = 0.79$; $Chi^2 = 178.03$, df = 8 ($P < 0.00001$); $I^2 = 96\%$
Test for overall effect: Z = 2.57 (P = 0.01)

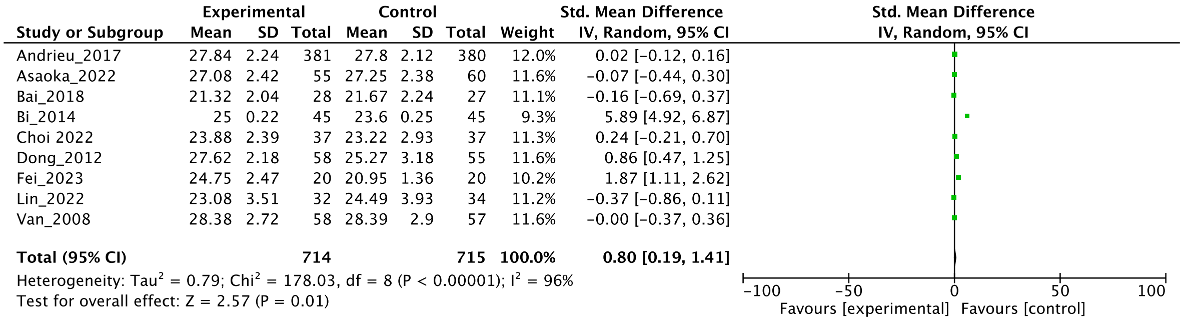

Fig. S7 Forest plot of the pooled effect estimate using MMSE
