# Supplementary material for: Effects of dietary supplements on cognitive outcomes and physiological biomarkers in mild cognitive impairment: a systematic review and network meta-analysis
Source: Front Nutr. 2026 Apr 29;13:1775177. doi: 10.3389/fnut.2026.1775177 (PMC13167511; doi:10.3389/fnut.2026.1775177)
Supplement: Supplementary file 3 [file Presentation_2.PDF]

1. PubMed

(("mild cognitive impairment"[Title/Abstract] OR MCI[Title/Abstract] OR "cognitive impairment"[Title/Abstract] OR "cognitive decline"[Title/Abstract] OR "Cognitive Dysfunction"[MeSH Terms])
AND
("Diet"[MeSH Terms] OR diet[Title/Abstract] OR "dietary intervention"[Title/Abstract] OR "nutritional supplement"[Title/Abstract] OR vitamin[Title/Abstract] OR mineral[Title/Abstract] OR "omega-3"[Title/Abstract] OR antioxidant[Title/Abstract] OR "Mediterranean diet"[Title/Abstract] OR "MIND diet"[Title/Abstract] OR "Diet Therapy"[MeSH Terms] OR "Dietary Supplements"[MeSH Terms] OR "Nutritional Physiological Phenomena"[MeSH Terms]))
AND
("Randomized Controlled Trial"[Publication Type] OR "Controlled Clinical Trial"[Publication Type] OR randomized[Title/Abstract] OR placebo[Title/Abstract] OR "Clinical Trials as Topic"[MeSH Terms] OR randomly[Title/Abstract] OR trial[Title/Abstract])

2.Embase

('mild cognitive impairment'/exp OR 'cognitive impairment'/exp OR 'cognitive dysfunction'/exp OR 'mild cognitive impairment':ti,ab OR MCI:ti,ab)
AND
('nutritional supplement'/exp OR 'vitamin'/exp OR 'mineral'/exp OR 'omega-3 fatty acid'/exp OR 'antioxidant'/exp OR 'Mediterranean diet'/exp OR 'MIND diet'/exp OR 'nutritional supplement':ti,ab OR vitamin:ti,ab OR mineral:ti,ab OR 'omega-3':ti,ab OR antioxidant:ti,ab OR 'Mediterranean diet':ti,ab OR 'MIND diet':ti,ab)
AND
('randomized controlled trial'/exp OR 'controlled clinical trial'/exp OR randomized:ti,ab OR placebo:ti,ab OR trial:ti,ab)
AND
('cognition'/exp OR 'cognitive function':ti,ab OR memory:ti,ab)
AND
[humans]/lim

3. CINAHL

(MH "Cognitive Impairment" OR MH "Mild Cognitive Impairment" OR TI "mild cognitive impairment" OR AB "mild cognitive impairment" OR TI MCI OR AB MCI OR TI "cognitive impairment" OR AB "cognitive impairment" OR TI "cognitive decline" OR AB "cognitive decline")
AND
(MH "Nutrition" OR MH "Diet" OR MH "Diet Therapy" OR MH "Dietary Supplements" OR TI nutrition OR AB nutrition OR TI diet OR AB diet OR TI "dietary intervention" OR AB "dietary intervention" OR TI "nutritional supplement" OR AB "nutritional supplement" OR TI vitamin OR AB vitamin OR TI mineral OR AB mineral OR TI "omega-3" OR AB "omega-3" OR TI antioxidant OR AB antioxidant OR TI "Mediterranean diet" OR AB "Mediterranean diet" OR TI

"MIND diet" OR AB "MIND diet")
AND
(MH "Clinical Trials" OR MH "Randomized Controlled Trials" OR TI randomized OR AB randomized OR TI placebo OR AB placebo OR TI trial OR AB trial)

4. PsycINFO

(DE "Cognitive Impairment" OR DE "Mild Cognitive Impairment" OR TI "mild cognitive impairment" OR AB "mild cognitive impairment" OR TI MCI OR AB MCI OR TI "cognitive impairment" OR AB "cognitive impairment" OR TI "cognitive decline" OR AB "cognitive decline")
AND
(DE "Nutrition" OR DE "Diet" OR DE "Diet Therapy" OR DE "Dietary Supplements" OR TI nutrition OR AB nutrition OR TI diet OR AB diet OR TI "dietary intervention" OR AB "dietary intervention" OR TI "nutritional supplement" OR AB "nutritional supplement" OR TI vitamin OR AB vitamin OR TI mineral OR AB mineral OR TI "omega-3" OR AB "omega-3" OR TI antioxidant OR AB antioxidant OR TI "Mediterranean diet" OR AB "Mediterranean diet" OR TI "MIND diet" OR AB "MIND diet")
AND
(DE "Clinical Trials" OR DE "Randomized Controlled Trials" OR TI randomized OR AB randomized OR TI placebo OR AB placebo OR TI trial OR AB trial)

5. Cochrane Library

("mild cognitive impairment" OR MCI OR "cognitive impairment" OR "cognitive decline")
AND
("nutrition" OR "diet" OR "dietary intervention" OR "nutritional supplement" OR "vitamin" OR "mineral" OR "omega-3" OR "antioxidant" OR "Mediterranean diet" OR "MIND diet")
AND
("randomized controlled trial" OR randomized OR placebo OR trial)

6. Web of Science

TS=("mild cognitive impairment" OR MCI OR "cognitive decline" OR "cognitive dysfunction" OR "cognitive impairment")
AND
TS=("nutrition" OR "diet" OR "dietary intervention" OR "nutritional supplement" OR "vitamin" OR "mineral" OR "omega-3" OR "antioxidant" OR "Mediterranean diet" OR "MIND diet")
AND
TS=("randomized controlled trial" OR "randomized" OR "controlled trial" OR placebo OR trial)
